# Cross Reality (XR): Challenges and Opportunities Across the Spectrum

**DOI:** 10.1007/978-3-030-58948-6_4

**Published:** 2021-03-12

**Authors:** Cindy Ziker, Barbara Truman, Heather Dodds

**Affiliations:** 1 Pennsylvania State University, Altoona, PA USA; 2 Valdosta State University, Valdosta, GA USA; 3 Ziker Research, San Jose, CA USA; 4 University of Central Florida, Orlando, FL USA; 5 Independent Researcher, New York, NY USA

## Abstract

Cross Reality (XR) resources hold promise for enhancing instruction and learning experiences in and out of the classroom. Appropriate XR applications can provide the foundation for new types of learning environments and experiences while bringing users together to create unique communities of inquiry and practice. Here we explore the opportunities and benefits of harnessing the affordances of XR while exploring the challenges associated with implementation. Recommendations and implications for future research are also addressed.

## Emerging Trends and Pedagogies

Cross Reality (XR) refers to a group of emerging technologies such as virtual reality (VR), augmented reality (AR), and virtual worlds (VWs) that involve the use of 3D models/simulations across physical, virtual, and immersive platforms. The path to optimizing the use of XR in education is not always easy to navigate. However, with adequate support, XR has the potential to help faculty and students transcend the boundaries of the classroom by providing new types of environments for presenting and delivering instructional content and creating learning experiences with the power to develop unique communities of inquiry and practice.

Throughout this chapter, we will explore scenarios populated by a typical future student named Andi and share examples of her engagement with Cross Reality throughout her educational career. These scenarios are designed to illustrate what lies ahead in education, as these technologies become more and more ubiquitous, allowing students like Andi to move seamlessly within the Reality-Virtuality Continuum.

### Definitions

For the purpose of this chapter, Cross Reality or XR refers to technologies and applications that involve combinations of mixed reality (MR), augmented reality (AR), virtual reality (VR), and virtual worlds (VWs). These are technologies that connect computer technology (such as informational overlays) to the physical world for the purposes of augmenting or extending experiences beyond the real. Especially relevant to the definition of XR is the fact that this term encompasses a wide range of options for delivering learning experiences, from minimal technology and episodic experiences to deep immersion and persistent platforms. The preponderance of different terms for slightly different technologies indicate that this is a growth area within the field. Here we provide a few definitions of these technologies.

MR—Mixed reality refers to a blend of technologies used to influence the human perception of an experience. Motion sensors, body tracking, and eye tracking interplay with overlaid technology to give a rich and full version of reality displayed to the user. For example, technology could add sound or additional graphics to an experience in real time. Examples include the Magic Leap One and Microsoft HoloLens 2.0. MR and XR are often used interchangeably.

AR—Augmented reality refers to technology systems that overlay information onto the real world, but the technology might not allow for real-time feedback. As such, AR experiences can move or animate, but they might not interact with changes in depth of view or external light conditions. Currently, AR is considered the first generation of the newer and more interactive MR experiences.

VR—Virtual reality, as a technological product, traces its history to approximately 1960 and tends to encompass user experiences that are visually and auditorily different from the real world. Indeed, the real world is often blocked from interacting with the virtual one. Headsets, headphones, haptics, and haptic clothing might purposely cut off all input except that which is virtual. In general, VR is a widely recognizable term, often found in gaming and workplace training, where learners need to be transported to a different time and place. VR experiences in STEM often consist of virtual labs or short virtual field trips.

VW—Virtual worlds are frequently considered a subset of VR with the difference that VWs are inherently social and collaborative; VWs frequently contain multiple simultaneous users, while VRs are often solo experiences. Another discrimination between virtual reality and virtual worlds is the persistence of the virtual space. VR tends to be episodic, with the learner in the virtual experience for a few minutes and the reality created within the experience ends when the learner experience ends. VWs are persistent in that the worlds continue to exist on computer servers whether or not there are active avatars within the virtual space (Bell 2008). This discrimination between VR and VW, however, is dissolving. VR experiences can be created to exist for days, and some users have been known to wear headsets for extended periods of time. Additionally, more and more VR experiences are being designed to be for game play, socialization, or mental relaxation. The IEEE VR 2020 online conference and the Educators in VR International Summit 2020 offered participants opportunities to experience conference presentations in virtual rooms as avatars while interacting with presenters and conference attendees (see Sect. 2.5 for more information).

Relevant to defining VWs, Correia et al. (2016) conducted a meta-analysis on the potential of using virtual worlds for learning and training (p. 407), while Mann et al. (2018) proposed a different definition of XR with variations of real and synthetic applications that make up their Multimediated Reality Continuum (p. 12), for another term. According to Mann et al. (2018, abstract), “As a new field of study, All Reality is multidisciplinary. We must consider not just the user, but also how the technology affects others, e.g. how its physical appearance affects social situations, and how sensor-based reality (e.g. wearable and implantable cameras in the smart city) affects privacy, security, and trust. All Reality includes Virtual Reality (VR), Augmented Reality (AR), X-Reality (XR), X-Y Reality (XYR), and Mixed, Mediated, etc. realities (MR).”

CVEs—Collaborative virtual environments are communication systems in which multiple interactants share the same three-dimensional digital space despite occupying remote physical locations (Yee and Bailenson 2006).

Embodiment—Embodiment is defined by Lindgren and Johnson-Glenberg (2013) as the enactment of knowledge and concepts through the activity of our bodies within an MR (mixed reality) and physical environment (p. 445). Embodiment can also be experienced as a suspension of disbelief while using avatars (digital individual representations) in a fully online virtual world. In fact, embodiment can be experienced as group phenomena that may lead to the development of communities of practice (CoP). Truman (2014) studied the relationship of embodiment in collaborative virtual environments (CVEs) and its reflexive properties of the primary avatar (learner/user) related to the theoretical framework of transdisciplinarity (p. 59). Attachment to avatars resulting in embodiment was not always found, suggesting that some individuals may be incapable of embodied experience (Truman 2014: 232).

The deeply immersive nature of some forms of XR has had a powerful effect in studies of multifaceted empathy. Manipulating the full environment around a learner, including sight, sound, smell, taste, pressure, heat, and texture promises to be significant in impact. “VR feels real, and its effects on us resemble the effects of real experiences. Consequently, a VR experience is often better understood *not as a media experience, but as an actual experience,* with the attendant results for our behavior” (Bailenson 2018: 46). Studies have shown that learners can quickly adopt an avatar as a personal representation of their own bodies; this effect is known as body transfer. When learners accept a digital object as a real object, the Proteus effect (Yee and Bailenson 2007) has been achieved, and manipulations of the digital object are biochemically accepted as real (Fox et al. 2016). Each of these defined forms of technology, MR, AR, VW, and XR, provides learners with real experiences.

### Key Trends

XR is becoming ubiquitous across society in domains such as entertainment, healthcare, government, military, education, and industry training for manufacturing and automation. Three key trends that are influencing the adoption of XR include:

1. The entertainment sector’s leadership in promoting societal acceptance of immersive applications drive down the consumer costs for equipment used in virtual, mixed, and augmented reality. Examples include Nomadic that offers team-based, immersive gaming in a physical environment (Nomadic 2019) and The Void, created by the Walt Disney Company (Disney, 2019). When consumers have access to trying XR applications in a rich-media context without having to buy first, it is predicted that accelerated adoption will occur for campus and home-based uses.
2. Pervasive use of XR is leading to the creation of new forms of partnerships and the potential for mass collaboration. More sustainable open-source software communities are examples of collaborations that build ecosystems for virtual world platform development. University involvement in developing the XR ecosystem has been largely ad hoc. Potentially, if universities coordinate to design and develop communities of practice using XR, standards will develop faster for interoperability, building a robust ecosystem. The Advanced Distributed Learning (ADL) Initiative (2019), an organization of the US Department of Defense (DoD), provides analogous support for interoperability, reusability, open standards, and architecture. The most recent developments include the Total Learning Architecture (TLA) that includes the Competency Management System (CaSS), learner modeling for analytics development, e-learning standards, and the Sharable Content Object Reference Model (SCORM). DoD innovations funded and supported by ADL must meet security requirements.
3. The combination of sensors (Internet of Things), 2D virtual (web) environments, 3D immersive environments, and virtual experiences is a global phenomenon representing massive, rapid change that will impact society. When Industry 4.0 or the Fourth Industrial Revolution (4IR) combines with the third wave of the Internet (Internet of Everything), XR will be poised for ubiquitous use across society (Dindar et al. 2009: 34). The World Economic Forum identified 12 core areas that make up 4IR. These are “big data, artificial intelligence (AI), and internet of things (IoT), virtual and augmented realities, additive manufacturing, blockchain and distributed ledger technology, advanced materials and nano-materials, energy capture, storage and transmission, new computing technologies, biotechnologies, geoengineering, neurotechnology, and space technologies” (Lieu et al. 2018: 2753).

Taking these trends into consideration, the following section explores XR learner activities with respect to STEM content and instructional design.

## Use of XR in 2026

During a 2018 X-FILEs Workshop, educators, researchers, and professionals gathered to discuss and hypothesize about the future of XR within STEM higher education. Ideas generated from this workshop formed the basis for this chapter.

### Content Presentation

Faculty and instructors typically think about instructional content to present, chunk, and sequence in their courses. Incorporating XR for graded assignments requires departmental and ideally institutional support to make the most of purchasing and design decisions. Appropriate XR applications can provide the foundations for new types of learning environments and experiences. XR can also bring users together creating new communities of inquiry and practice. With adequate support, XR can help users transcend the boundaries of classroom and web-based instruction. XR applications are discussed in this section in terms of the context of faculty demands and institutional resources based upon variables of size, staffing, need to scale, and emphasis on research focus. Taxonomies, toolkits, and means to obtain data valuable for creating baseline analytics using XR all support scholarship and research regardless of institution type.

#### Opportunities

XR presents an opportunity for an entirely new approach to learning design that can leverage the principles of transmedia learning. Transmedia learning provides a guide for educational experiences in XR, primarily because it offers the learner a real or simulated world with extra layers of overlaid information. As the learner is often at the center of these XR experiences, transmedia learning builds upon creating stories across experiences and devices where the learner is the hero possessing self-determination and self-regulation. The use of game-based learning, serious games, and gamification is included under Raybourn’s (2018) transmedia learning framework, which provides heuristics for designing and personalizing transmedia learning. According to Raybourn (2018), “The transmedia learning framework (TLF) is meant to provide ways to think outside the box about learning, or what we normally consider education. The TLF is a way to employ new media in a way that is going to augment your learning experience, to include engaging yourself at the neurological level” (Raybourn 2018: 1).

#### Challenges

Authoring 3D content has become easier, but is not easy enough for fast production by most users and especially faculty. Library leadership is needed to track 3D open educational resources and provide authority for managing data into existing information schema and standards. Staff support for matchmaking of needs and content will require significant commitments of time to experiment and play with XR possibilities. Partnering with innovators on campus already doing XR can result in valuable informal professional development offerings that include panel discussions, hands-on demos, and tracking of effective uses.

#### Implementation Strategies

Instructors should be very clear about why they are selecting XR learner activities as opposed to other modes of teaching and learning. Apparent gains in learning, or increased learner preference, by utilizing XR can be attributed to the novelty effect (Clark 1985) if compared to non-robust, non-immersive learning choices. Aldrich (2009) suggests that higher interactive virtual environments (HIVE) do work as learning activities because immersive activities stimulate emotional involvement, which is necessary for learning to move from working memory to long-term memory.

#### Research Questions

Future research relevant to content preparation in XR should address the following:

- What are the rights and ethical decisions that need to be made within organizations dedicated to XR development?
- How can common interface usage be created to apply to the design of XR experiences? For example, Control-C means copy in multiple software programs.
- What heuristics can be developed to increase understanding and outcomes of which XR application is most appropriate for various learning situations?
- How can XR facilitate collaboration on distributed team-based projects that are part of real systems used in society?
- How can incorporation of XR augment tutoring, counseling, help desks, or other virtual interactions?

### Interactions and Communications

#### Opportunities

Some of the early evidence that interactions with XR applications are effective comes from corporate training (Maxwell and McLennan 2012), which suggests that adult learners experience enhanced transfer, possibly due to situated learning and because such training often has an immediate real-world application. This point should not be lost; Adult learners appear to be the first to have larger-scale learning success when focusing on the application of learning.

Collaborative virtual environments in the workplace allow for co-design and co-development to embed scenarios or situational problem-solving questions into immersive 3D environments. Microsoft’s HoloLens 2 is innovating in the workplace environment by bringing what they call “instinctual interaction” into XR use (Upload 2019). The company is currently expanding into the workforce market, hoping to tap up to two billion frontline workers with HoloLens (Weise 2019).

Researchers are still in the nascent stages of discovering how the power of XR can be optimized. While studies by Yee and Bailison and others have examined the role that XR activities can play in behavioral skills like eliminating negative stereotyping, fostering empathy, and increasing scientific inquiry thinking processes, research on the concept of mirror neurons as the conduit for behavioral skills is maturing past conceptual to actual stages (Rizzolatti and Craighero 2004; Fox et al. 2016). Early data is pointing to the fact that the brain believes that XR activities really happen and as such, the internal biochemical changes of learning are the same as the real experience (Bailenson 2018). For example, XR can provide the benefits of field trips without requiring travel. Further XR ideas include: manipulating space and time; such as looking inside of a supernova; using expensive lab equipment at little cost; and scenarios as yet unimagined that are too dangerous or difficult. Thus XR can bring a new range of experiences to the learners of tomorrow.

For health, XR can also have a positive impact in the form of aiding cancer treatments (Chirico et al. 2016). The arrival of the COVID-19 pandemic and the closing of contact sports are causing a rise in the use of eSports. If, following the mirror neuron research from Rizzolatti and Craighero (2004), the mind believes that what the eyes see is actually happening, it is logical to assume that engaging in eSports could have a positive impact on physical health. During eSports, the body is being flooded with the same biochemicals as traditional athletes. Increased physical health could be measured with two blood pressure tests, one at each end of an academic semester for eSports athletes.

Research findings on empathy studies using XR are mixed at this time. Herrera et al. (2018) found that there are positive long-term impacts via VR experience on the topic of homelessness. However, a VR experience with color-blindness resulted in no short-term empathy increases. In some cases, exposure to VR decreases empathy. Companies like Equal Reality (https://equalreality.com/) are using virtual reality for diversity training as a way to impact perspectives on privilege and power.

#### Challenges

XR platforms also have significant accessibility concerns that need to be addressed with both hardware and software. Many experiences require full 360° movement and motion controller input and do not have any accessibility features built in to allow for a wider variety of users. There are a variety of ways that this affects users with a range of abilities (Ryan 2019). Opportunities to create compelling social XR applications are exciting but must not come at the cost of alienating users.

When we introduce multiple users into an XR experience, we introduce the possibility for a whole host of issues that are not encountered in single user educational experiences. We encounter significant issues related to safety, accessibility, and privacy that must be accounted for very early on in the design process of any multi-user educational experience (Reilly et al. 2014).

When using existing social virtual reality experiences, female users reported struggling “in a social sense, dealing with strangers and encountering behaviors that they equated to real life scenarios where they had experienced harassing behavior. In the social VR worlds they visited, they encountered flirting, a lack of respect for personal boundaries, socially undesirable behavior and in the end, most were not interested in using these platforms to meet strangers” (Outlaw and Duckles 2017). The social problems we deal with in location-based educational environments follow us into XR. Outlaw and Duckle’s study of women in social VR experiences highlights the issues of personal and social safety faced by those who experience a lack of social safety outside of VR. In VWs, the phenomenon known as griefing could negatively impact digital systems in the form of attacks that reduce digital commerce (Bakioglu 2009).

#### Implementation Strategies

Educators should be looking for ways to leverage existing tools to create meaningful interactions using XR technologies while continuing to explore new opportunities that have not yet been developed. “Remote real-time collaboration with socio-technical systems and dialogue tools aimed at promoting collaborative learning and deepening the space of debate and producing epistemic interactions is in the interest of designers, engineers and educators around the globe. This calls for enabling more platforms for real-time collaboration between teams and networks” (Wendrich et al. 2016). XR technologies will also provide new forms of leadership engagement between students, faculty, and institutions.

Using commercially available applications, like Altspace or Bigscreen, educators already have the opportunity to present more traditional digital content (video, photos, presentations, etc.) to students in an XR environment. Utilizing these types of systems allows for a low barrier to entry that requires minimal customization or onboarding. Educators can quickly adapt existing content to be used.

For universities that already have existing XR equipment, labs, and studios, there are great opportunities to invite students deeper into the design process and to create collaborations with other universities so students can interact with other students who might have similar access. It should be a top priority for institutions with these resources to leverage them, in order for students to connect with their peers at other universities in XR experiences.

#### Research Questions

Future research regarding interactions and discussions using XR should address the following:

- How can XR improve peer-to-peer learning?
- How do people construct their own virtual identity?
- How do XR technologies differ in their effectiveness for fostering meaningful discussions?
- What factors cause learners from different backgrounds to be inhibited in multi-user XR experiences?

### Learner Activities with XR

Prior to 1910, STEM classes were taught primarily with direct instruction (Olson and Loucks-Horsley 2000). This started to change with John Dewey’s emphasis on active learning activities that occurred outdoors, outside the science classroom. Over time, however, STEM teachers wanted activities that would be active in cognitive load but could also be done indoors to eliminate problems of weather, distance, and availability (Brown 2003). The space-era Commission on Science Education of 1964 has strongly influenced STEM instruction for the past half-century by requiring the inclusion of experiential learning approaches, known as laboratory courses (labs), in addition to rote learning of the scientific method (Livermore 1964). Virtual labs provide equal learning gains when compared to in-person labs (Faulconer and Gruss 2018). The emphasis in XR, however, is less on content knowledge and more on inquiry scientific thinking processes and behaviors and then collaborating and communicating. Consequently, VR, VW, AR, and all forms of XR have a history of providing the valid and immersive active learning activities required within STEM domains (Nelson and Ketelhut 2007).

#### Opportunities

Designing with XR requires a fundamental shift to a Human-Centered Design philosophy that involves putting human needs, capabilities, and behavior first (Jerald 2018: 15). XR provides the opportunity to experience just-in-time *immersive*, experiential learning that uses concrete yet exploratory experiences involving senses that result in lasting memories. Here we discuss opportunities for social applications with XR.

XR learner activities are usually created for individual use, which may or may not need to be simultaneously experienced as a class together at the same time or place with the instructor. Activities can be designed into instruction with VR headsets, high-resolution screens, smartphones, or other solo technological devices for use inside and outside of the classroom. Feedback is also often individualized (Lynch and Ghergulescu 2017). Multiple characteristics contribute to the growing ubiquity of XR.

STEM XR learner activities often have these characteristics:

- Decreased danger to learners, increased respect for the environment, and replicability—chemicals and radiation are virtual and can be used indefinitely (Faulconer and Gruss 2018). Inside VR, “mistakes are free” (Bailenson 2018: 24). In virtual reality, chemicals can be mixed in the wrong order and cleaned up just by pressing a recycle button (Faulconer and Gruss 2018). Surgical emergencies can be practiced with no ill effects upon real patients (Health Scholars n.d.).
- No limits to time or space—learners can control time within a biological system (Clark 2009), or learners can go to the International Space Station. There can be full instructor control of the virtual experience, important when engaging in dangerous or trauma-replicating activities (Bailenson 2018). XR has the power to make difficult science concepts to learn easier by “making the unseen seen” (Potkonjak et al. 2016: 4).
- Increased respect for learner ethics and accessibility needs—virtual dissections reduce need for organisms, and text and sound can be added as layers of additional information, that information can be more thorough upon demand, and XR resources paused, reset, and can be available anytime (Lynch and Ghergulescu 2017).
- Decreased cost due to low maintenance and the ability to easily replicate the experience for more learners (Faulconer and Gruss 2018).

Some published examples of the use in XR learner activities within STEM include:

- Genome Island within the VW Second Life provides asynchronous learning experiences where time can be accelerated and multiple generations of organisms can be studied within a few minutes (Clark 2009).
- Labster provides virtual labs that go beyond simple bench work and include narratives that show the role of collecting samples, safety protocols, and analyzing results (Pate 2020).
- Arizona State University partnered with Smart Sparrow to make “immersive, interactive virtual field trips” (Mead et al. 2019: 2) that allow for instantaneous movement around a constructed paleoenvironment.
- Functional analysis of historic architectural structures to understand engineering innovation of buildings. Notre Dame is an example (CBS Interactive Inc. 2019).
- Learners can construct their own representation of their knowledge. The app Orb uses AR to allow students to create simple 3D objects that appear in real space (Donally 2018).
- Restivo et al. (2014) found that the use of AR in teaching direct current circuits allowed learners to have the ability to overlay real-world and real-time lab experiences.

#### Challenges

The technological challenges of the use of XR in learner activities should be acknowledged. There will always be first-use hesitation from learners, and they may tend to prefer more traditional activities until the newer technological interface becomes intuitive (Waldner et al. 2006). VR head-mounted displays can be burdensome with tethering cables and tight-fitting headsets, and if technology is shared or passed from learner to learner, there are the added challenges of the sharing of germs and body moisture. Further developments already coming in VR equipment include the use of temperature, pressure, and scent which can potentially disturb the learner. Bailenson aptly identifies the VR dangers of poor behavioral modeling, simulator sickness, eyestrain, and reality blurring (2018). VW can be addictive and can contain significant non-educational activities not suitable for young learners. AR can cause distractions if used walking down the street or while driving.

XR learner activities should be designed to increase accessibility to all learners by utilizing the XR strengths of multi-layered information display. However, some learners will be held back from full XR activity by visual, physical, and social abilities such as stroke, vertigo, epilepsy, or age-related reaction time. It should also be noted that the encompassing nature of VR headsets might create some discomfort or danger for any learners as they can no longer fully see and control their body and body space.

Keeping young learners focused while using technology can be a challenge. Tallyn et al. (2005) found that AR could bring media-based learning to life when used in concert with paper-based learning as an instructional theme through a learning experience. Learners focused on working through a worksheet or workbook could reap the benefits of AR while not going off-track with their learning.

#### Implementation Strategies

Combining VR applications such as virtual worlds with other XR applications can serve as a multiplier effect for research where communities of practice span across international boundaries. User support communities are robust in the virtual worlds of Second Life and OpenSimulator where institutional representation often occurs at the grassroots level. Immersive conferences such as the Virtual Worlds Best Practices in Education and the OpenSimulator Community Conference enable sharing of R&D across XR applications. Network members in organizations such as the Immersive Learning Research Network and Educators in VR overlap the disciplines of education, technology, and industry in order to further the research-based effective application of XR technologies.

Examining how augmented reality and virtual reality social systems differ, how they may become blended, and the necessary design considerations, we can see how as these technologies converge there will be new possibilities for exploration and collaboration (Miller et al. 2019). Already there are social XR platforms, like AltspaceVR and Bigscreen, that allow users with access to a variety of XR devices to share virtual spaces and content from their own devices with each other.

In juxtaposition to the experiences of users in 3D worlds using 2D screen, or those using video-based telepresence systems, XR social platforms allow for users to feel as if their “virtual self is experienced as the actual self” (Aymerich-Franch et al. 2012). This sense of social presence (Oh et al. 2018) offers new areas of exploration for users of this technology. We can now consider not just adapting existing educational content, but conceiving of entirely new ways of educating that leverage an embodied experience and allow for a huge range of augmentation.

The affordances of XR environments include opportunities for geographically dispersed learners to learn in an environment similar to their traditional classrooms without forfeiting the ability to learn at their own pace and in their own time zone (Olasoji and Henderson-Begg 2010). Computer-supported collaborative work (CSCW) or team-based projects involve looking at the individual and collaborative potentials based on individual and shared contributions. Ward and Sonneborn (2009) note that methods of assessing creative problem-solving in groups will need to include measures of how people personalize their learner group contributions and the effect that such individualized collaboration has on the quantity and quality of ideas produced.

#### Research Questions

Future research in learner activities using XR should include:

- What learner activities in XR are active learning approaches, as opposed to passive approaches?
- How are learner activities in XR fostering critical STEM skills including inquiry, empathy, collaboration, and communication?
- How do XR learning activities impact human physiological and anatomical structures of the brain, such as for improving self-regulated learning with bio-feedback from usage of wearables tracked by avatars?
- How can XR learning activities foster play as a foundation for learning?

- *Since her earliest school years, Andi has been going on virtual field trips that utilize basic VR headsets in the classroom and immersive rooms at museums. Her initial experiences involved little interaction and were more look-and-see experiences, such as taking a field trip to the Great Wall of China while using a VR headset. Gradually, Andi’s teachers added interactive experiences during which Andi learned to navigate controllers to draw, assemble, and bounce virtual objects in small group activities with her classmates. By middle school, Andi was a confident presenter in virtual reality platforms and often played social VR games outside of school. In 6th grade, Andi wrote a report on her virtual visit to Mars, ending with her resolution to become an astronaut.*
- *In high school, Andi enjoyed participating in eSports and played on an all-female team with the potential to win $30,000 in college scholarships. She also engaged in weekend virtual science workshops on biofuel creation sponsored by corporations looking to increase students’ scientific collaboration skills of observation* *, cause-and-effect monitoring, and communication of results. Andi regularly joined virtual check-ins with scientists at international museums and connected virtually with astronauts at the National Aeronautics and Space Administration and the International Space Station.*

### Assessment

#### Opportunities

The immersive nature of XR has the potential to provide engaging assessment experiences that represent real-world activities more accurately than traditional paper and pencil measures. Still, assessment of learning in virtual environments typically employs the use of external instruments that are administered outside of the virtual experience. This review examined the literature related to the types of assessments used in the context of XR, with a focus on methods for assessing skills, evaluating work products and performance, and analyzing log files.

Conventional assessments, such as pre and post, multiple-choice items, short answer items, and open-ended questions, are frequently used to assess the effectiveness of learning in a virtual environment. Ketelhut et al. (2006) created a pre-post, self-designed content test to assess knowledge of science inquiry and process skills during an investigation of a science curriculum, implemented through a virtual environment called River City. Labster’s HMD and desktop VR lab experiments include embedded multiple-choice questions and a point system to track students’ scores as they progress through experiments. Metrics, such as assessing the number of questions asked in a lab experience within and without a pre-lab XR experience, offer additional possibilities for collecting performance measures.

In contrast, evaluating learner-created work products developed directly within immersive virtual environments provides opportunities for assessing authentic learner performance. Rose (1995) leveraged the psychological theories of information processing and constructivism to identify specific approaches for measuring learning in VR that included performance tasks, such as world building, problem-solving, and the evaluation of the quality of final products. Olasoji and Henderson-Begg (2010) studied a Second Life course that required learners to produce a summative assessment containing a scientifically accurate depiction of a biological molecule or bioinformatic concept. Work products such as these can be evaluated to produce data concerning the learner’s performance through a rubric, a scoring guide, or an automated scoring procedure (Mislevy et al. 2017).

The Virtual Performance Assessment (VPA) Project , created by the Harvard Graduate School of Education, is an open-ended 3D immersive virtual environment designed for performance assessments of science inquiry skills in multiple virtual scenarios. Students engage in authentic inquiry activities and solve scientific problems by navigating around the virtual environment as avatars, making observations, interacting with non-player characters (NPCs), gathering data, and conducting laboratory experiments (Baker and Clarke-Midura 2013). VPA enables the automated and non-intrusive collection of process data (event logs or logged actions and behaviors) and product data (students’ final claims), facilitating the capture and assessment of science inquiry in situ (Jiang et al. 2015). These examples represent advancements toward relevant methods for assessing learning within virtual settings.

A frontier of educational assessment is the development of automated methods of evaluating log files, through which cognitively meaningful patterns and features of work are detected and characterized as observations (Mislevy et al. 2017). The dynamic nature of the computer system allows recording of learner interactions and data gathering in the background as the learner moves through an XR experience (Rose 1995). Log file analysis of this type of data is often referred to as stealth assessment, which has been used to measure problem-solving and spatial skills (Shute et al. 2015), assess causal reasoning in the World of Goo (Shute and Kim 2011), and assess systems thinking in Taig Park (Shute et al. 2010). An advantage of stealth assessments is that data can be collected without disrupting learner flow within the XR experience (Shute et al. 2015).

#### Challenges

Assessments in XR present several challenges that can impact the integrity of the inferences that can be made from results. Examples include interference due to headset discomfort, lack of familiarity with navigating the virtual world, and gaps in alignment between the assessment method and the content being assessed. Construct-irrelevant variance caused by distractions in the virtual environment can negatively influence learner behavior. In multi-user environments, issues around trust, security, and identity can arise, given that users can create multiple accounts and avatars (Warburton 2009).

In the context of stealth assessments, the complexity of log file coding schemes can make analysis difficult, while a lack of aligned external validation instruments can pose additional reliability challenges (Wang et al. 2015). According to Wang et al., “If a researcher wants to create stealth assessments within an existing commercial game, the first step is to make sure that either the coding in the log files is simple enough to understand, or the coding scheme is available from the game developer so that changes can be made to the information that is being captured” (2015: 5). It is important to note that not every logged interaction or tracked gaze represents evidence of learning. Validating accurate scoring systems for virtual activities that have the ability to predict performance in real-world settings presents a formidable challenge for XR assessment developers.

#### Implementation Strategies

Implementing assessments that have the capacity to elicit evidence of what learners know and can do requires close alignment between learner interactions and the expected outcomes (Code et al. 2012). By leveraging the power of evidence-centered design (ECD) (Mislevy and Haertel 2006), assessment developers can create an evidentiary assessment argument supported by a conceptual assessment framework that includes task specifications, evaluation procedures, and measurement models. In a virtual environment, provisions for accommodations and the use of the principles of universal design for learning (UDL) are critical for ensuring accessibility and equity during assessment implementation.

#### Research Questions

Overall, the development of reliable assessment methods within XR is still in the early stages. Future research is needed to examine the following research questions:

- What indicators will inform whether contextual learning using XR is connected to curriculum alignment?
- What are the most effective and useful means of tracking competency for various skills?
- What is the efficacy of novel forms of assessment within XR?
- How can learning and performance be accurately assessed in XR environments?
- What methods of assessment are possible in XR environments?

- *To mitigate safety risks during Andi’s first XR experiences, her teachers limited the viewing to under 2 min. Teacher assistants served as spotters, and the space for engaging with the hardware was cleared of hard surfaces. Alternative experiences were set up in 2D for any learner.*
- *One of Andi’s teachers wisely included safety precautions in her XR choices and picked a platform where the students were not tracked and did not need to log-in with authentic credentials. When the learner’s session ends, the entire session is deleted from the host’s servers. At the high school computer lab, the instructional technology specialists set up all of the VR devices to be controlled and monitored from a main station and ensured that devices could not be accessed outside of the school network without specific teacher permission. Andi’s instructors selected learning experiences where teleportation was used rather than abrupt motion, to reduce incidences of motion* *sickness.*
- *Routinely, XR devices were disinfected daily during the school year. Learners were taught to wipe down the headset and controllers before using them. All parents were informed of the planned instructional use of XR through the year, and opt-in signatures were requested.*
- *Andi’s early choices within XR environments were limited to looking at a spot, clicking on something, a small range of “right click” alternative choices, and click and drag. As she gained confidence, she navigated multiple screens (e.g., in XR and in browser) at the same time, learned fly commands, combined virtual objects* *, and panned around 3D depictions. Due to products like Unity and other 3D content creators, Andi was able to create unique XR experiences with new textures and objects. Her instructors used her content creations within XR as evidence of learning, rather than relying on traditional forms of assessment outside of XR.*

### Co-curricular Activities 

Here we explore the experience of learners in the context of academic endeavors beyond classroom interactions that include socialization, employment, and responsibilities that provide opportunities, challenges, and potential for future research using XR. Testing and adoption of commercial XR applications by learners enables instructors to harness new data that is useful for providing analytics of learner performance while supporting motivation to engage in STEM. The National Academies of Sciences, Engineering, and Medicine (2016: 95) reported that the use of co-curricular programming can affirm students’ self-perceptions of competence to mitigate impacts of a stigmatizing STEM academic culture.

#### Opportunities

The pervasive use of XR is leading to the creation of new forms of partnerships and the potential for mass collaboration. More sustainable open-source software communities are examples of collaborations that build ecosystems for virtual world platform development. Further expansion of these collaborations is recommended to advance the field of XR.

**Fig. 1:**
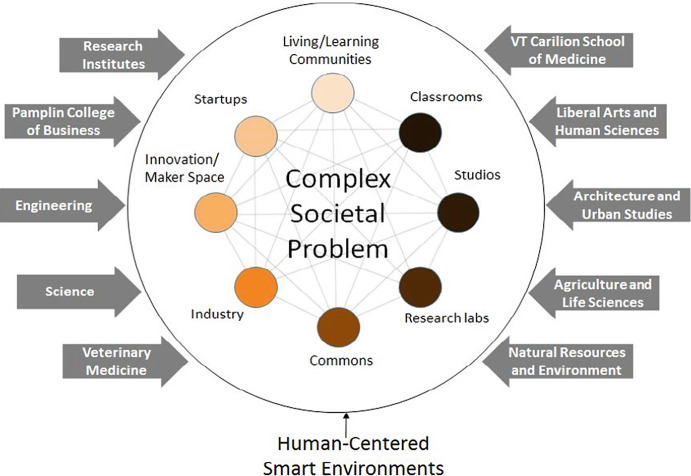
Hundley (2016: 8) integrated innovation hubs

Obtaining value from the use of XR applications requires new interdisciplinary collaboration across departments, campuses, and partnerships with industry to manage complexity in pursuit of improved learning outcomes. Virginia Tech created a report for envisioning the campus of the future where organization and reorganization will be required among stakeholders including the greater community to fuse intellectual and co-curricular life as Human-Centered Smart Environments (HCSE). Figure 1 illustrates a vision for technology-enabled, interdisciplinary participation in societal needs that create new forms of learning opportunities. The emphasis in this diagram is that collaboration from multiple sources is necessary in human-centered environments . This collaboration needs to be made and remade in successive cycles in order for the positive impacts to be effective. For example, maker-spaces and research efforts on college campuses need to collaborate with industry to make the results widely available.

Virginia Tech’s HCSE plans include Living Laboratories that are “... experiential environments wherein students and researchers can engage in learning, discovery, and innovation through interactions with real-world systems and communities. Living labs incorporate ‘smart’ autonomous systems that range from furniture and wearable technology to buildings and the surrounding landscape. These systems will assist humans in multiple ways that include supporting wellness, collaboration, solitude, inspiration, and disruption” (Hundley 2016: 11). The Virginia Tech HCSE model represents the framework for a type of immersive, digital learning commons that can incorporate existing instructional and information software systems, designed for the most common XR applications used across the institution.

#### Challenges

The field of XR is in need of the creation of standards and norms for implementing technologies in educational settings, which could provide support for and accelerate their adoption by higher education institutions. XR applications currently do not have universal standards that enable interoperability and seamless integration into existing software. Professional associations such as the IEEE’s IC Industry Consortium on Learning Engineering (ICICLE) (https://www.ieeeicicle.org/) XR working groups (https://www.ieeeicicle.org/xreality-sig) are investigating how to make the user experience more seamless and transparent. Potentially, if universities coordinate to design and develop communities of practice using XR, standards will develop faster for interoperability, building a robust ecosystem. The pervasive use of XR is leading to the creation of new forms of partnerships and the potential for mass collaboration. More sustainable open-source software communities are examples of collaborations that build ecosystems for virtual world platform development. Further expansion of these collaborations is recommended to advance the field of XR.

#### Implementation Strategies

Recommendations for higher education institutions and faculty seeking to advance the use of XR in co-curricular instruction include:

- Promoting interdisciplinary connections (ways to encourage and support different ways/lenses of looking at issues or problems) such as learner-produced content and authoring. Examples of this approach include student hackathon events and leadership engagement with clubs and organizations.
- Expanding access to virtual internships and field trips.
- Broadening collaborations across academics, industry, and community (making connections) including collaborating with the military for bootstrapping research and development.
- Increasing access to industry and government facilities and tools using XR.
- Creating standards and norms to support implementation.

During the COVID-19 pandemic, educational institutions of all sizes implemented XR as a social connection strategy. Platforms such as Discord, Mozilla Hubs, AltspaceVR, and VirBELA which were previously used only for co-curricular meetings and game spaces became more popular. The power of these platforms was leveraged to conduct virtual global conferences, such as the Immersive Learning Research Network 2020 Conference, the IEEE VR 2020 Conference, and the Educators in VR 2020 Conferences. These events engaged thousands of students, educators, and industry practitioners from around the world in virtual convenings that demonstrated how XR resources can be used for the purpose of sharing knowledge and professional networking, when traditional conference formats are impossible.

*As a college senior, Andi has experienced several courses in virtual worlds and designed her dorm room using the Walmart AR app to make furniture choices. Training for her current job has included virtual simulations* *that address complex problems through global collaborations and negotiations among individuals and large groups in virtual settings. She regularly attends virtual conferences that connect her with thousands of attendees in virtual spaces, such as Altspace, Mozilla Hubs, and VirBELA*. *Utilizing industry and media contacts, Andi can include any of her family and friends in XR activities because she can find XR resources that work for a range of ages and abilities. Many XR experiences have text, as well as icon labels so that Andi can engage with learners that need language-flexible choices. Andi’s father often engages in VR training before he visits a new building site, while her mother uses XR training to practice empathy skills with her fellow emergency medical technicians. For Andi and her family, XR has become a common part of daily life.*

#### Research Questions

Recommendations for areas of future research related to co-curricular XR include the following research questions:

- What are the mental, physical, and neurological effects of using XR technology on the learner for various durations (including long-term)?
- Which XR learning activities increase time on learning tasks?
- How can the effective use of XR technologies improve collaboration practices for transdisciplinary scholarship and research?
- How does XR support improvement among individuals and teams, especially at a distance?
- How do institutions leverage XR to create communities of practice to promote cross-institutional, cross-industry, and cross-domain collaboration of R&D?

## Conclusions

As posited in the chapter introduction, the influence of the entertainment sector, new collaborations between technology and business, and the ubiquity of the Internet of Things all indicate that the required technology for XR might already be in American homes, classrooms, and workplaces in the form of smartphones, computers, or game systems. “VR alone is expected to reach $60 billion in 2020” (Bailenson 2018: 9), and AR is expected to reach $60 billion in 2020 (Porter and Heppelmann 2017). With the COVID-19 pandemic, institutions of all sizes are considering the incorporation of XR experiences.

In conclusion, while there are many opportunities for students and faculty who are able to leverage the benefits of XR to enhance coursework, a variety of obstacles may hinder scaling. Optimizing the use of XR in higher education requires the support and resources of an interdisciplinary community of committed professionals from education, government, and industry who will work together with researchers to overcome the existing challenges that limit adoption. The development of common standards could advance this effort. There is also a need to invest in future research regarding the implementation and the assessment of the effectiveness of XR on learning. Technological and practical solutions are possible through the collaboration of experts and the financial investment for research in this field.
